# WNT signaling and distant metastasis in colon cancer through transcriptional activity of nuclear β-Catenin depend on active PI3K signaling

**DOI:** 10.18632/oncotarget.1626

**Published:** 2014-02-19

**Authors:** Steffen Ormanns, Jens Neumann, David Horst, Thomas Kirchner, Andreas Jung

**Affiliations:** ^1^ Institute of Pathology, Ludwig Maximilians Universität, Munich, Germany; ^2^ German Cancer Consortium (DKTK); and German Cancer Research Center (DKFZ), Heidelberg, Germany

**Keywords:** WNT, β-Catenin, PI3K, colon cancer, metastasis

## Abstract

We determined whether active PI3K signaling together with nuclear accumulation of β-Catenin is necessary to fully activate canonical WNT signaling and examined the association of both signaling pathways with colon cancer progression. Using reporter gene assays we examined the activation of β-Catenin mediated transcription upon PI3K inhibition with or without β-Catenin nuclear accumulation. Ectopically induced as well as constitutively active WNT signaling strictly required PI3K activity whereas PI3K inhibition had no effect on β-Catenin subcellular localization but impaired β-Catenin binding to WNT target gene promoters and decreased WNT target gene expression. Transcriptional activity of nuclear β-Catenin depended on active PI3K signaling as nuclear accumulation of β-Catenin failed to induce WNT reporter gene transcription upon PI3K inhibition. PI3K dependend transcriptional transactivation of β-Catenin relies on events beyond phosphorylation at the AKT target site serine 552, as S552D-phosphomimetic β-Catenin mutants were unable to restore WNT signaling when inhibiting PI3K. To study the prognostic value of PI3K pathway activation (activating *PIK3CA* mutations or loss of PTEN expression) and nuclear β-Catenin expression, both variables were determined in 55 matched pairs of primary right sided colon cancer cases with or without distant metastasis. Activating mutations in the *PIK3CA* gene or loss of PTEN expression did not correlate with distant metastasis while high nuclear β-Catenin expression combined with activation of the PI3K pathway identified cases in which distant metastasis had occurred. Activation of the PI3K pathway was not associated with nuclear β-Catenin expression. We conclude that the transcriptional activity of nuclear β-Catenin depends on PI3K activity. However, PI3K on its own does not affect β-Catenin subcellular localization. Both factors synergize for full WNT signaling activity and are associated with distant metastasis in colon cancer. Thus, the detection of high nuclear β-Catenin expression and simultaneous PI3K pathway activation identifies colon cancer patients with a high risk for distant metastasis.

## INTRODUCTION

Colon cancer (CC) is one of the most prevalent human malignancies, causing more than 50.000 deaths per year in the United States [1]. Prognosis mainly depends on disease recurrence and distant metastasis after potentially curative tumor resection [2]. Most CC harbor genetic alterations in key elements of the canonical WNT signaling cascade [3], mainly in APC or β-Catenin, leading to hyperactivation of this signaling pathway. In the nucleus, β-Catenin functions as a transcription factor, driving the expression of various target genes which are implicated in the hallmarks of cancer, such as epithelial to mesenchymal transition (EMT) and metastasis [4, 5].

Although the same APC or β-Catenin mutations are present in all tumor cells of a CC, the WNT pathway is not uniformly active in these tumors. Instead, high WNT activity and nuclear β-Catenin expression often are confined to the tumor margin, reflecting an intratumoral WNT signaling heterogeneity [6,7]. This phenomenon has been termed the “β-Catenin paradox” and led to the discovery that other signaling pathways may modulate WNT signaling activity in CC with APC or β-Catenin mutations [8]. Hepatocyte growth factor signaling and mitogen-activated protein kinase (MAPK) signaling have been identified as such modulators and may be important contributors to WNT mediated tumor progression [9-12].

Another pathway that may intersect with WNT signaling in this context is Phosphatidylinositide-3-kinase (PI3K) which transmits signals from transmembrane growth factor receptors to transcription factors. Enhanced PI3K signaling, either through loss of expression of its antagonist PTEN, or through activating mutations in exons 9 and 20 of the *PIK3CA* gene, encoding the catalytic subunit p110α of PI3K, can be observed in a variety of human malignancies, including colon cancer [13] [14]. Moreover, a link between WNT and PI3K signaling was observed in non-colonic cell lines, where PI3K signaling could activate β-Catenin mediated transcription, through β-Catenin phosphorylation by AKT [15]. PI3K also forms part of a signaling cascade involving Rac1 and JNK, which downstream of LRP5/6 receptors controls the nuclear localization of β-Catenin in ST2 murine stromal cells [16]. Additionally, inhibition of PI3K-AKT signaling reduced WNT signaling in medulloblastoma cells [17]. We therefore hypothesized that PI3K signaling may be an additional essential modulator of WNT activity, especially in colon cancer.

According to this hypothesis, we here provide mechanistic and clinicopathological evidence that active PI3K signaling is mandatory for full WNT pathway activation, β-Catenin mediated target gene transcription, and tumor progression of CC. Importantly, we demonstrate that the nuclear localization of β-Catenin and its transcriptional transactivation are independent processes, linked by PI3K. Based on these findings, we propose a two-step-model of β-Catenin mediated transcription, in which its nuclear accumulation and subsequent transcriptional transactivation by PI3K are two fundamental steps for WNT signaling activation, driving colon cancer progression.

## RESULTS

### Pharmacological inhibition of PI3K suppresses β-Catenin transcriptional activity

**Figure 1:**
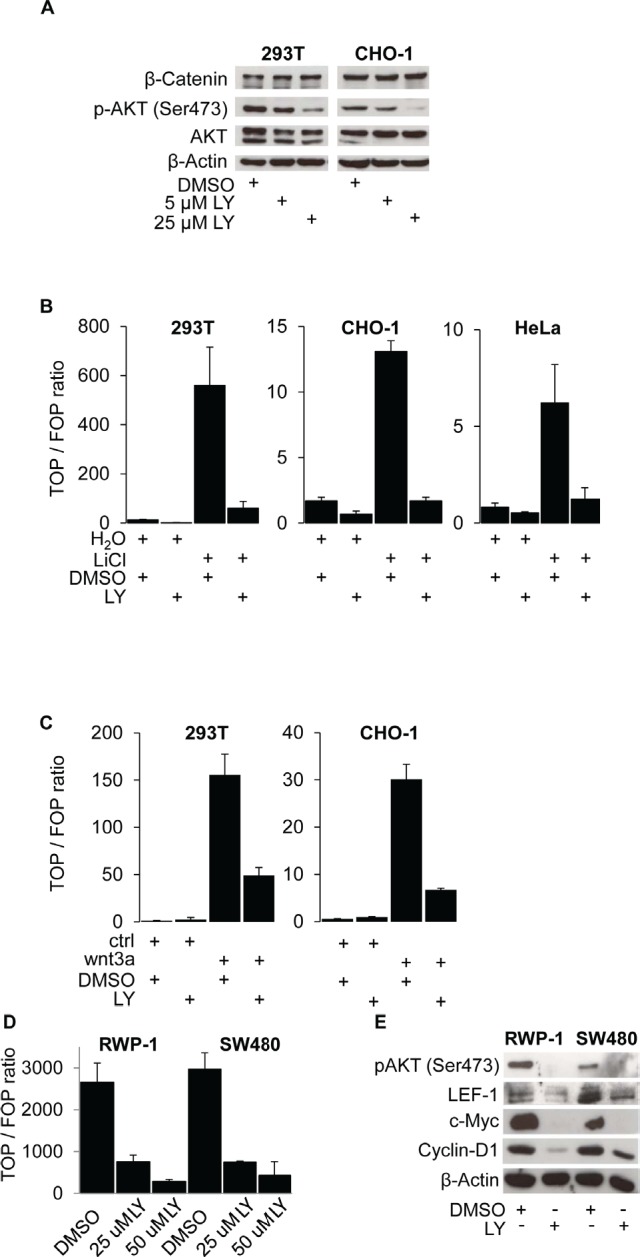
PI3K activity is required for β-Catenin mediated transcription and WNT target gene expression (A) Western blot analysis of 25 μg whole cell protein lysates of 293T and CHO-1 cells treated with 25 μM LY294.002 (LY) or DMSO control for 12 hours. (B) TOP-flash luciferase reporter assay of 293T, CHO-1 and HeLa cells treated with 30 mM LiCl and / or 25 μM LY and the respective vehicles for 12 hours after transfection. (C) TOP-flash luciferase reporter assay of 293T and CHO-1 cells treated with wnt3a conditioned medium (wnt3a) or control conditioned medium (ctrl) and 25 μM LY or vehicle for 12 hours after transfection. (D) TOP-flash luciferase reporter assay of SW480 and RWP-1 cells treated with the LY concentrations indicated or vehicle for 12 hours after transfection. (E) Western blot analysis of 25 μg whole cell protein lysates of RWP-1 and SW480 cells treated with 25 μM LY for 12 hours.

To determine whether PI3K activity is required for β-Catenin transcriptional activation, we used 293T and CHO-1 cells, both of which are devoid of constitutively activating WNT pathway mutations. In these cell lines, we ectopically stimulated WNT signaling using LiCl [18] and wnt3a-conditioned medium [19] and then treated these cells with the PI3K specific inhibitor LY294.002 (LY) [20]. As anticipated, PI3K inhibition dose dependently reduced protein levels of phospho-AKT (Ser473), which served as readout for PI3K activity. However, protein levels of total AKT and notably β-Catenin remained unchanged (Figure 1 A). To test the functional effect of PI3K inhibition, we then used TOP-flash luciferase reporter assays to gauge β-Catenin transcriptional activity in 293T, CHO-1 and HeLa cells. Following addition of LiCl (Figure 1 B) or treatment with wnt3a-conditioned medium (Figure 1 C), we observed a strong induction of β-Catenin transcriptional activity which could efficiently be blocked by inhibiting PI3K with LY. Next, we used SW480 and RWP-1 cancer cells which carry APC-mutations and show constitutive WNT pathway activation [21], [22]. Also in these tumor cells, PI3K inhibition dose dependently reduced β-Catenin transcriptional activity (Figure 1 D) and readily reduced the expression of the WNT target genes c-MYC [23], Cyclin-D1 [24] and notably LEF-1 [25] (Figure 1 E). These observations indicate that active PI3K signaling is required for β-Catenin transcriptional activity.

LY is also known to inhibit Casein kinase 2 (CK2) in vitro [26], which may influence on β-Catenin transcriptional activation [27]. To exclude possible off-target effects of LY treatment, we examined whether the observed changes in β-Catenin transcriptional activity were indirectly mediated through CK2 inhibition. Therefore, we monitored CK2 activity through phosphorylation of CdC37, a well known target of CK2 [28]. In Western blots of LY-treated 293T and CHO-1 cell lines, no change in phospho-CdC37 (Ser13) expression was observed (supplementary figure S1 A). In contrast, the specific CK2 inhibitor 4,5,6,7-tetrabromobenzotriazole (TBB) [29] only reduced phospho-CdC37 levels at high concentrations under conditions of serum starvation (supplementary figure S1 B). These data show that suppression of WNT signaling through LY is not mediated by effects on CK2.

### WNT signaling through nuclear β-Catenin depends on PI3K activity

**Figure 2:**
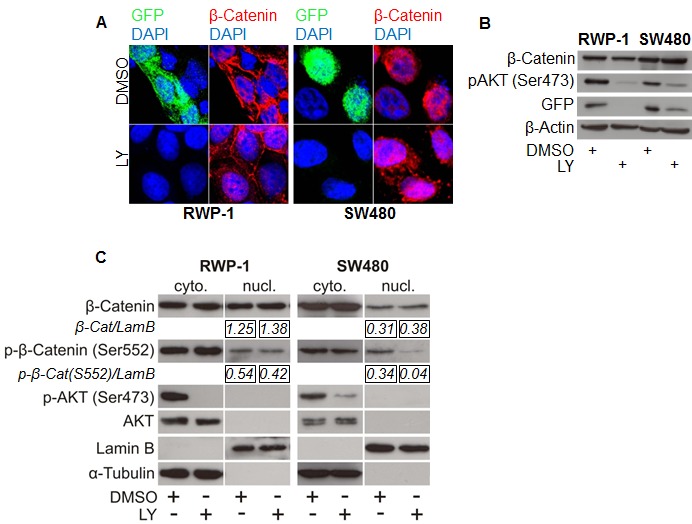
PI3K inhibition reduces β-Catenin mediated transcription without affecting β-Catenin subcellular localization (A) Immunofluorescence microscopy of RWP-1 and SW480 cells transfected with pTOP-GFP reporter plasmid and treated with 25 μM LY or vehicle (DMSO) for 12 hours, stained for β-Catenin and GFP. DAPI marks nuclei. (B) Western blot analysis of 25 μg whole cell protein lysates of the remaining RWP-1 and SW480 cells from experiment (A). (C) Western blot analysis of 15 μg protein of nuclear and cytoplasmic enriched fractions of RWP-1 and SW480 cells treated with 25 μM LY or vehicle (DMSO) for 12 hours. Relative signal densities of nuclear β-Catenin and phospho-(S552)-β-Catenin expression compared to the loading control Lamin B are indicated in the boxes below each image.

To understand the influence of PI3K signaling on WNT activity and nuclear β-Catenin on a cellular level, we treated cancer cells with LY and monitored WNT activity using TOP-GFP reporters and β-Catenin immunofluorescence. Interestingly, LY treatment significantly reduced GFP fluorescence and thus β-Catenin mediated transcription while, unexpectedly, the subcellular localization of β-Catenin remained unaffected as β-Catenin was found to be expressed equally in the nucleus and the cytoplasm (Figure 2 A). We confirmed these results in Western blot analyses on the protein level using whole cell lysates (Figure 2 B) as well as cytoplasmic and nuclear enriched protein fractions (Figure 2 C). These data implicated that PI3K activity is required for β-Catenin transcriptional activity and that these effects are not solely mediated through changes in β-Catenin subcellular localization.

**Figure 3:**
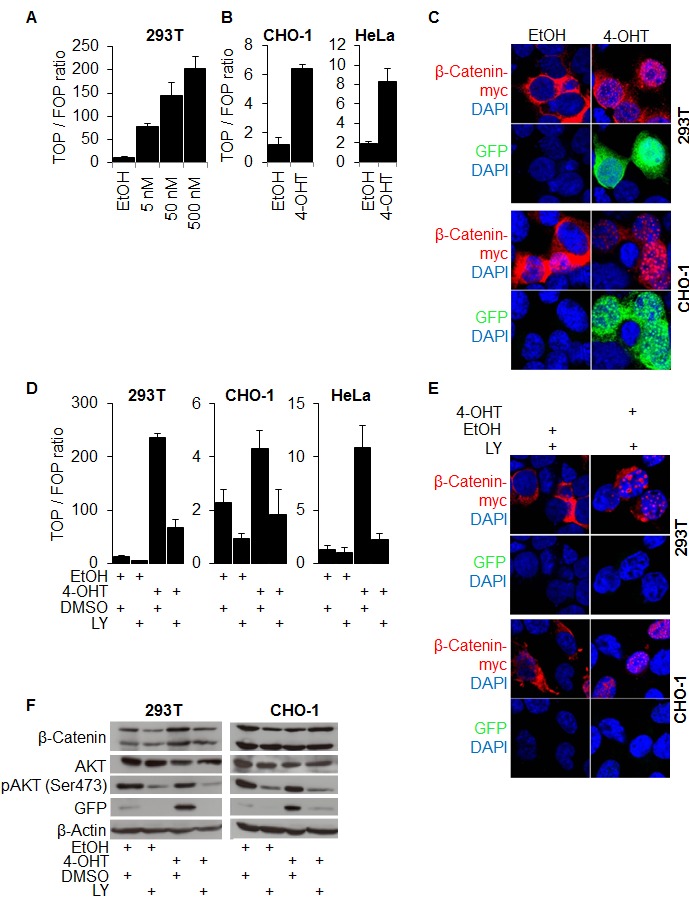
β-Catenin nuclear accumulation and transcriptional transactivation by PI3K are required for β-Catenin mediated transcription (A) TOP-flash luciferase reporter assay of 293T cells expressing a stabilized mutant β-Catenin protein (Δ45-β-CateninER-myc) whose nuclear accumulation is inducible by 4-OH-tamoxifen (4-OHT) treatment. Cells treated for 12 hours with the 4-OHT concentrations indicated or vehicle (EtOH). (B) TOP-flash luciferase reporter assay of CHO-1 and HeLa cells expressing Δ45-β-CateninER-myc treated for 12 hours with 500 nM 4-OHT (C) Immunofluorescence microscopy of 293T and CHO-1 cells expressing Δ45-β-CateninER-myc treated with 4-OHT or vehicle for 12 hours. DAPI marks nuclei. (D) TOP-flash luciferase reporter assay of 293T, CHO-1 and HeLa cells expressing Δ45-β-CateninER-myc treated with 500 nM 4-OHT or vehicle and 25 μM LY or vehicle. (E) Immunofluorescence microscopy of cells transfected with Δ45-β-CateninER-myc and pTOP-GFP, treated with 25 μM LY and 500nM 4-OHT or vehicle for 12 hours. DAPI marks nuclei. (F) Western blot analysis of 25 μg whole cell protein lysates of 293T and CHO-1 cells transfected with Δ45-β-CateninER-myc and pTOP-GFP as in figure (E) and treated with the indicated substances for 12 hours.

To investigate if the nuclear import of β-Catenin may happen independently from its transcriptional activation, we fused a myc-tagged modified estrogen receptor (ER-myc) [30] to a degradation resistant mutated β-Catenin (Δ45-β-Catenin), resulting in the fusion protein Δ45-β-CateninER-myc. Adding the ER ligand 4-hydroxy-tamoxifen (4-OHT) to 293T, CHO-1, and HeLa cells expressing this construct significantly activated the transcription of TOP-flash reporter in a dose dependent manner (Figures 3 A, B). Also, strong nuclear accumulation of β-Catenin, induced upon 4-OHT addition, co-localized with high β-Catenin mediated transcription as monitored through TOP-GFP (Figure 3 C). Vehicle treated control cells showed a cytoplasmic localization of β-Catenin and no or weak GFP fluorescence (Figure 3 C). In this system, forced nuclear β-Catenin accumulation thus activated β-Catenin mediated transcription. We then treated these cells expressing the Δ45-β-CateninER-myc fusion protein with LY and monitored β-Catenin mediated transcription through both TOP-flash and TOP-GFP reporters. It turned out that PI3K inhibition significantly suppressed reporter activity, even if nuclear accumulation of β-Catenin was forced through 4-OHT treatment (Figure 3 D, E).

Finally, we confirmed these observations by examining the expression levels of β-Catenin, GFP and phospho-AKT (Ser473) in Western blot analyses. Indeed, following 4-OHT addition, we found a strong induction of β-Catenin mediated transcription as indicated by high GFP expression. This was significantly reduced by inhibiting PI3K signaling with LY. Remarkably, LY treatment even reduced basal β-Catenin mediated transcription, while β-Catenin expression levels were unaffected (Figure 3 F).

**Figure 4:**
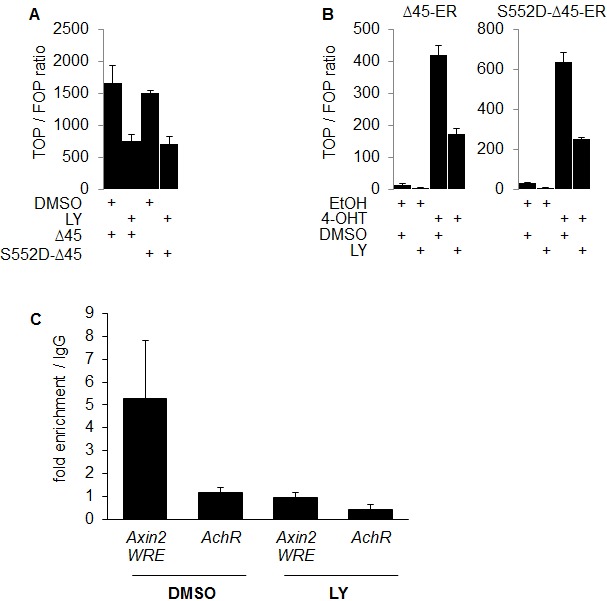
PI3K activity is required for β-Catenin transcriptional transactivation beyond serine 552 phosphorylation and increases β-Catenin binding to WNT target gene promoters (A) TOP-flash luciferase reporter assay of 293T cells transiently expressing Δ45-β-Catenin (Δ45) or phosphomimetic mutant Δ45-β-Catenin-S552D (S552D-Δ45) treated with 25 μM LY or vehicle for 12 hours. (B) TOP-flash luciferase reporter assay of 293T cells transiently expressing Δ45-β-CateninER-myc (Δ45-ER) or phosphomimetic mutant Δ45-β-Catenin-S552D-ER-myc (S552D-Δ45-ER) treated with 500 nM 4-OHT or EtOH and 25 μM LY or vehicle for 12 hours. (C) Quantitative PCR analysis of immuno precipitated chromatin (ChIP) of SW480 cells treated with 25 μM LY or vehicle for 12 hours, using mouse anti-β-Catenin antibody or control mouse normal immunoglobulin G1 (IgG1). Values represent fold enrichment of anti-β-Catenin antibody precipitated DNA over control IgG1 precipitated DNA. Human achetylcholine receptor promoter, which is devoid of WNT responsive elements served as control region.

Upon PI3K inhibition, we observed a siginificant decrease in the nuclear expression of β-Catenin phosphorylated at the putative AKT phosphorylation site serine 552 in SW480 cells and a minor decrease in RWP-1 cells (Figure 2 C). Thus, we tested if the expression of a degradation resistant mutant β-Catenin protein carrying a phosphomimetic aspartate at amino acid residue 552 would be able to restore WNT signaling when inhibiting PI3K using LY. As expected, overexpression of the stabilized Δ45-β-Catenin readily induced strong expression of the luciferase reporter gene in TOP-flash assay, which could efficiently be blocked by LY. No difference in signaling reduction through PI3K inhibition was observed when overexpressing phosphomimetic S552D-Δ45-β-Catenin in 293T cells (Figure 4 A). To further investigate a possible effect of PI3K inhibition on the signaling properties of nuclear β-Catenin, we experimentally induced the nuclear accumulation of phosphomimetic S552D-Δ45-β-CateninER-myc using 4-OHT and tested its ability to induce WNT signaling upon PI3K inhibition in TOP-flash reporter asssays. Phosphomimetic S552D-Δ45-β-CateninER-myc was unable to restore the induction of reporter gene expression when inhibiting PI3K, even in the context of forced nuclear accumulation (Figure 4 B). With these findings in mind, we addressed if β-Catenin would still be able to bind to target gene promoters when inhibiting PI3K. Thus we employed chromatin immunoprecipitation (ChIP) using an anti-β-Catenin antibody in LY and vehicle treated SW480 cells. LY treatment significantly reduced β-Catenin binding to the WNT responsive element (WRE) of the WNT target gene *Axin2* as shown by decreased enrichment of *Axin2* WRE in β-Catenin immunoprecipitated chromatin compared to immunoprecipitated chromatin using control IgG1 (Figure 4 C).

Collectively, these results indicate that both the nuclear accumulation of β-Catenin and simultaneous PI3K activity are required for β-Catenin-mediated transcription. PI3K inhibition does not affect nuclear shuttling, but reduces β-Catenin binding to target gene promoters. Moreover, these data suggest that transcriptional activity of nuclear β-Catenin requires transactivation by PI3K signaling beyond serine 552 phosphorylation.

### Activation of the PI3K pathway with concurrent nuclear accumulation of β-Catenin correlates with distant metastasis in colon cancer

**Figure 5:**
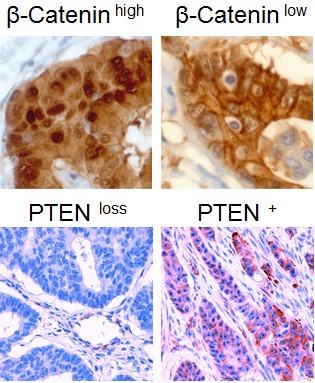
Immunohistochemical staining of colon carcinomas using anti-β-Catenin antibody (400-fold magnification) or anti-PTEN antibody (200-fold magnification) Exemplary cases of high or low nuclear β-Catenin expression and PTEN expression or PTEN loss are displayed respectively.

We previously demonstrated that high nuclear β-Catenin expression in CC is associated with tumor progression and metastasis [31]. To assess whether PI3K modulated WNT signaling might contribute to this observation, we analyzed PI3K signaling and β-Catenin expression in a well defined matched pair case control collection of right sided colon cancers with and without synchronous liver metastasis (supplementary table S2 A). As previously reported [31], tumors showing strong nuclear β-Catenin expression, suggestive of high WNT signaling activity, were more likely to have metastasized than tumors lacking strong nuclear β-Catenin expression (table 1 A; 71.4 % vs. 40.0 %; p=0.002). In contrast, high PI3K activity alone, as assessed through loss of PTEN expression [32] (Figure 5) or presence of activating mutations in the exons 9 or 20 of the *PIK3CA gene* (supplementary figure S3), showed no significant association with distant metastasis (table 1 A; p=0.815, p=0.329). However, when examining the combination of PI3K signaling and nuclear β-Catenin, a significant effect of activated PI3K signaling on distant metastasis was observed: All cases (100%) with PTEN loss or *PIK3CA* mutation and high nuclear β-Catenin expression had metastasized, whereas of the *PIK3CA* wildtype tumors with normal PTEN expression and high nuclear β-Catenin expression, only 20.0 and 61.5% showed metastasis (table 1 B; p=0.001, p=0.003). This association became even clearer and statistically stronger when analyzing *PIK3CA* mutational status and PTEN expression status together as regulated or deregulated PI3K pathway (table 1 C). These findings indicate a strong effect of PI3K signaling on tumor progression associated WNT signaling.

**Table 1:** (A) High nuclear β-Catenin expression correlates with distant metastasis in colon cancer Univariate correlation analysis of distant metastases with nuclear β-Catenin expression, PTEN expression and PIK3CA mutational status, analyzed in the matched case control collection. **(B) High nuclear β-Catenin expression and concurrent activating PIK3CA mutation or loss of PTEN expression correlate with distant metastasis in colon cancer**. Univariate correlation analysis of distant metastases with nuclear β-Catenin expression combined with the PTEN expression or PIK3CA mutational status. **(C) High nuclear β-Catenin expression with concurrent constitutive activation of the PI3K pathway correlate with distant metastasis in colon cancer**. Univariate correlation analysis of distant metastases with nuclear β-Catenin expression combined with the regulatory status of the PI3K pathway. Regulated means PTEN expression and wildtype PIK3CA gene, deregulated means loss of PTEN expression and/or presence of activating mutations in the PIK3CA gene. **(D) Activation of the PI3K pathway does not correlate with the nuclear accumulation of β-Catenin**. Univariate correlation analysis of the nuclear β-Catenin expression and the regulatory status of the PI3K pathway

A		M0 n (%)	M1 n (%)	p-value				
nuclear β-Catenin	low	45 (60.0)	30 (40.0)	0.002				
	high	10 (28.6)	25 (71.4)					
PIK3CA gene	wt	43 (49.4)	44 (50.6)	0.815				
	mutated	12 (52.2)	11 (47.8)					
PTEN	expressed	36 (53.7)	31 (46.3)	0.329				
	lost	19 (44.2)	24 (55.8)					

B	PIK3CA gene	M0 n (%)	M1 n (%)	p-value	PTEN	M0 n (%)	M1 n (%)	p-value
low nuclear β-Catenin	wt	33 (55.0)	27 (45.0)	0.001	expressed	26 (63.4)	15 (36.6)	0.003
	mutated	12 (80.0)	3 (20.0)		lost	19 (55.9)	15 (44.1)	
high nuclear β-Catenin	wt	10 (37.0)	17 (63.0)		expressed	10 (38.5)	16 (61.5)	
	mutated	0 (0.0)	8 (100.0)		lost	0 (0.0)	9 (100.0)	

C	PI3K pathway	M0 n (%)	M1 n (%)	p-value				
low nuclear β-Catenin	regulated	19 (59.4)	13 (40.6)	0.00027				
	deregulated	26 (60.5)	17 (39.5)					
high nuclear β-Catenin	regulated	10 (52.4)	9 (47.6)					
	deregulated	0 (0.0)	16 (100.0)					

	PI3K pathway							
D	regulated n (%)	deregulated n (%)	p-value					
low nuclear β-Catenin	55 (73.3)	20 (26.7)	0.089					
high nuclear β-Catenin	20 (57.1)	15 (42.9)						

Of note, loss of PTEN expression or *PIK3CA* mutations were not associated with increased metastasis in colon cancers with low nuclear β-Catenin levels (table 1 B and C), or with other variables, such as age, gender, nodal status, T category or tumor grade (supplementary table S4). Furthermore, we found no significant correlation of PTEN loss or *PIK3CA* mutations and high nuclear β-Catenin expression (table 1 D). These findings suggest that PI3K driven tumor progression depends on nuclear localization of β-Catenin, while PI3K activity alone is insufficient to induce β-Catenin nuclear translocation and WNT signaling.

Taken together, our *in vivo* results show that WNT driven colon cancer progression and metastasis depend on the coincidence of active PI3K signaling and nuclear β-Catenin expression.

## DISCUSSION

In this study, we identify PI3K signaling as a crucial modulator of WNT signaling activity. Using different cell lines with either wildtype WNT signaling components or pathway activating mutations in APC, we show that inhibition of PI3K signaling generally reduces WNT signaling by blocking β-Catenin-mediated transcription and the expression of the WNT target genes c-MYC, Cyclin-D1 and LEF-1. Although PI3K inhibition might also directly affect the expression of c-MYC and Cyclin-D1 [33], LEF-1 is known as a specific WNT target gene [34]. Our findings add to previous observations in which PTEN, a negative regulator of PI3K signaling, enhanced β-Catenin degradation, thus reducing WNT signaling activity [35]. Moreover, combined conditional knockout of PTEN and APC induced strong activation of both pathways, resulting in the formation of highly aggressive intestinal adenocarcinoma in mice, while loss of each factor alone did not lead to malignant tumorigenesis [36]. This provided a specific synergistic link between both pathways, as described for hematopoietic stem cells too [37]. Also, intestinal epithelial cells seemed to depend on PI3K signaling in host defense mechanisms against bacterial infections that require WNT signaling [38]. Our findings now demonstrate that strong exogenous WNT pathway activation by blocking GSK3-β activity with LiCl or direct stimulation with wnt3a could be abrogated through PI3K inhibition. Moreover, even in tumor cells with APC mutations, β-Catenin dependent transcription and expression of WNT target genes was compromised when blocking PI3K and this effect seems to be mediated by impaired binding of β-Catenin to its target gene promoters. Taken together, PI3K not only enhances and synergizes with WNT signaling but rather appears to be a necessary basis and general requirement for full β-Catenin mediated transcriptional activation. Importantly, when inhibiting PI3K, we excluded putative previously reported off target effects of this treatment through CK2 [27], providing additional certainty that our approach directly links PI3K and the WNT pathway.

The nuclear localization of β-Catenin is commonly regarded as being equivalent to active WNT signaling [8]. Whether shuttling of β-Catenin between the cytoplasm and the nucleus, for which several co-factors were identified [39, 16] is the only determinant of WNT target gene transcription is currently unclear, and our data shed useful light. PI3K inhibition reduced β-Catenin transcriptional activity, but surprisingly had no effect on the subcellular localization of endogenous β-Catenin. Instead, PI3K inhibition reduced the nuclear level of β-Catenin phosphorylated in the putative AKT phosphorylation site serine 552 [15]. Moreover, using a 4-OHT dependent system which allows for controlling of the subcellular localization of exogenous, degradation resistant β-Catenin, we demonstrate that transcriptional activation of β-Catenin strictly depends on PI3K signaling, even if β-Catenin is forced into the cell nucleus. Mechanistically, β-Catenin transcriptional activation in this context might rely on a direct phosphorylation of β-Catenin through PI3K dependent downstream effectors, such as AKT, as previously reported [15]. However, phosphomimetic S552D-β-Catenin mutants were unable to restore WNT signaling even in the context of a forced S552D-β-Catenin nuclear accumulation. We therefore reason that PI3K dependent transcriptional transactivation of β-Catenin relies on activating events beyond serine 552 phosphorylation, although it is possible that the phosphomimetic properties of an aspartate residue might not be sufficient to restore β-Catenin mediated transcription upon LY treatment. Based on these data, we imply a model in which, in a first step, WNT signaling through β-Catenin can be influenced through effects on its subcellular localization. In a second step, its transcriptional activity may additionally be modulated by PI3K signaling. The nuclear import of β-Catenin and its transcriptional transactivation thus appear to be independent processes. It remains to be investigated however, whether this model prevails under physiologic conditions in PI3K and WNT dependent tumors or tissues.

Because PI3K inhibition blocked WNT activation in APC mutated colon and pancreatic cancer cells, we propose an association of PI3K signaling and WNT signaling in CC and other malignancies. This is especially relevant regarding the fact that most CC carry mutations in APC [40] in all clonally derived tumor cells [41], suggestive of a constitutive and uniformly active WNT pathway in these tumors. Therefore, the existence of subpopulations of tumor cells with high and low WNT activity and the observation that nuclear accumulation of β-Catenin occurs in a minority of tumor cells at the invasive front of colorectal carcinomas denoted a paradox [6,8], which our data now helps to resolve. We demonstrate that PI3K signaling is required for high WNT signaling activity in APC-mutated colon cancer cells, thus providing a means of how external stimuli, e.g. from the tumor microenvironment, may modulate WNT signaling in colon cancer cells through differential activation of PI3K. Indeed, previous data showed induction of WNT activity together with putative stemness traits upon hepatocyte growth factor stimulation by tumor-surrounding myofibroblasts in colon cancer xenografts [9]. In a similar manner, we recently identified MAPK signaling as another regulator of WNT activity in CC [11], and both PI3K and MAPK may thus be central modulators of WNT signaling in colon cancers with APC-mutations and serve as the functional basis for heterogeneous WNT pathway activation in this tumor entity.

This link between PI3K and WNT signaling in colon cancers may eventually translate into clinical relevance. We and others previously associated the risk of colon cancer progression and the amount of tumor cells showing strong nuclear β-Catenin accumulation in CC [31], suggesting that tumor progression depends on tumor cells with high WNT signaling activity. It may be speculated whether this finding reflects putative stem cell characteristics [9,42] or EMT like phenotypes [43] of tumor cells with high WNT activity, driving disease progression. The assessment of direct PI3K downstream targets such as phospho-(Ser473)-AKT and phospho-(Ser552)-β-Catenin expression failed in our CC collection (data not shown), most likely due to poor tissue preservation through inefficient and heterogeneous fixation. Thus we chose to analyze PTEN expression and *PIK3CA* mutation status as reliable markers of PI3K activity. Here, we demonstrate in a well defined homogeneous study population, that stratifying colon cancers for activated PI3K signaling strongly enhanced the prognostic power of nuclear β-Catenin assessment. High expression of nuclear β-Catenin perfectly identified colon cancer cases with synchronous metastasis in cases with PTEN loss or activating *PIK3CA* mutations only, both indicative of active PI3K signaling [32,44]. In line with our *in vitro* data, we thus propose that PI3K signaling is crucial for WNT driven colon cancer progression in CC patients. Not only may the combined assessment of PI3K and WNT signaling thus serve as an improved prognostic tool identifying CC patients in need of intensified follow-up or adjuvant therapeutic strategies. It also opens a rationale for ongoing trials of PI3K pathway inhibitors, especially in colon carcinomas carrying a deregulated PI3K signaling axis [45]. Although controversial data has been published recently [46], there is vast preclinical data showing that PI3K signaling enhances metastatic potential and that PI3K pathway inhibition reduces metastatic dissemination of colorectal cancer cells carrying a mutated WNT pathway, which supports our findings [47-52]. Further studies are needed to address whether this effect is conducted through inhibition of WNT driven tumor progression and whether high WNT signaling activity in CC may help predict the therapeutic response to agents that target PI3K signaling.

## MATERIALS AND METHODS

### Cell culture and reagents

Cell lines were purchased from DSMZ (Deutsche Sammlung von Mikroorganismen und Zellkulturen), routinely screened for mycoplasma contamination and DNA typed to prove the identity of the cell lines used. Cells were cultured in DMEM / 10% FBS (fetal bovine serum), penicillin/streptomycin (Biochrom AG, Berlin, Germany) at 37°C, 5% CO2 in a humidified incubator and passaged before reaching confluence. The RWP-1 cell line was a kind gift from A.G. de Herreros (IMIM-Hospital del Mar and Universitat Pompeu Fabra, Barcelona, Spain). The wnt3a-producing L-cells and the corresponding control cells were a kind gift from M. Königshoff (Comprehensive Pneumology Center, Munich, Germany). Wnt3a-conditioned medium and corresponding control medium was prepared as previously described [19]. 4-Hydroxytamoxifen (4-OHT), 4,5,6,7-tetrabromobenzotriazole (TBB), 2-(4-morpholinyl)-8-phenyl-chromone (LY294.002, LY), lithium chloride (LiCl) and protein G coupled sepharose beads were purchased from Sigma. 4-OHT was dissolved in ethanol. TBB and LY294.002 were disssolved in DMSO. Monoclonal rabbit antibodies directed against pan-AKT (clone 11E7), phospho-AKT (Ser473) (clone D9E), GFP (clone D5.1), cdc37 (clone D11A3), LEF-1 (clone C12A5) and polyclonal rabbit antibodies directed against phospho-cdc37 (Ser13) and phospho-β-Catenin (Ser552) were purchased from Cell Signaling (Frankfurt a.M., Germany), mouse anti-β-Catenin antibody (clone 14) from BD Biosciences (Heidelberg, Germany), mouse-anti-β-Actin antibody (clone AC-74) and mouse anti-β-Tubulin (clone TUB 2.1) from Sigma (Munich, Germany) monoclonal mouse anti-myc tag antibody (clone 9E10) from Millipore (Schwalbach, Germany), monoclonal rabbit anti-c-MYC antibody (clone Y69) from Epitomics (Burlingame, CA, USA), monoclonal goat anti-Lamin B (clone M-20) and normal mouse IgG1 from Santa Cruz Biotechnology (SCBT, Santa Cruz, CA, USA) and monoclonal mouse anti-Cyclin-D1 antibody (clone DCS-6) from Neomarkers (Fremont, CA, USA). All transfections were done using Fugene 6 transfection reagent (Roche, Mannheim, Germany) according to the manufacturer's instructions. All other reagents were purchased from Sigma-Aldrich.

### Plasmids

The sequence encoding a stabilized deletion mutant β-Catenin (Δ45-β-Catenin) was amplified by PCR from pcI-neo-Δ45-β-Catenin (kind gift from B. Vogelstein, Johns Hopkins University, Baltimore, USA). The sequence encoding the tamoxifen sensitive mutant murine estrogen receptor (ER^TAM^) [30] was amplified by PCR from pBSKS-ER^TAM^ (kind gift from G. Evan, University of Cambridge, UK) adding a myc tag encoding sequence to its 3′end. Both PCR products were fused in frame using splicing by overlap extension PCR [53] and cloned into the *pcI-neo* backbone resulting in pcI-neo-Δ45-β-CateninER-myc. pTOP-Flash, pFOP-Flash and pRenillaTK plasmids were a kind gift of H. Clevers (Hubrecht Institute, Utrecht, The Netherlands). The pTOP-GFP plasmid resulted from replacing the luciferase gene in pTOP-Flash by the EGFP gene. The plasmids encoding stabilized phosphomimetic S552D-β-Catenin (S552D-Δ45-β-Catenin) or stabilized phosphomimetic S552D-β-Catenin fused to the mutant estrogen receptor (S552D-Δ45-β-CateninER-myc) were constructed using the QuikChange site-directed mutagenesis kit (Stratagene, La Jolla, CA, USA). Correct insertion of the mutations was tested by direct sequencing.

### Luciferase reporter assay

1×10^4^ cells were seeded in a 24-well plate and transfected with pTOP-Flash or pFOP-Flash respectively, together with pRenillaTK in a 1:10 ratio and the respective plasmid mentioned in the experiment as a total of 250 ng DNA using Fugene 6. After 4 hours, cells were treated as indicated. After 12 hours, dual luciferase reporter assays (Promega, Mannheim, Germany) were done according to the manufacturer's instructions using an automated Orion II luminometer (Berthold Technologies, Bad Wildbad, Germany). TOP / FOP ratios were calculated after normalizing luciferase values to renilla values. All experiments were done in triplicates and repeated at least twice. Representative results are shown.

### Immunoblotting

Cultured cells were washed within cold PBS, scraped and lysed in triple lysis buffer (Maniatis et al, 50 mM Tris-HCl, 150 mM NaCl, 0.02 % NaN_3_, 0.5 % Na-Deoxycholate, 0.1 % SDS, 1 % Nonidet P-40) containing 1 × PhoStop phosphatase inhibitor cocktail and 1 × Complete proteinase inhibitor cocktail (Roche) according to the manufacturer's instructions. After sonification for 3 seconds at 75% amplitude using an HTU Soni 130 sonifier (G. Heinemann, Schwäbisch-Gmünd, Germany), debris was removed by centrifugation for 20 min. at 20.000 g and 4°C. Nuclear and cytoplasmic enriched protein fractions were prepared as described previously [54]. Protein concentration was determined using DC protein assay kit (BioRad, Munich, Germany) following the vendors instructions. Equal amounts of protein were loaded on a discontinuous denaturing 10% polyacrylamide gel, electrophoretically separated and blotted onto PVDF membranes (BioRad). The membranes were incubated overnight at 4°C in the presence of the primary antibody diluted in TBS / 0.1 % Tween-20 containing 5% w/v non fat dry milk (anti-β-Actin antibody and anti-β-Tubulin antibody 1:10.000 dilution, all other antibodies 1:1000 dilutions). After washing and incubation with horseradish peroxidase (HRP) labeled secondary antibodies (1:10.000 dilution, Pierce, Rockford, IL, USA), blots were incubated with HRP substrate (Millipore, Darmstadt, Germany) and chemoluminescence was detected using x-ray films (GE Lifesciences, Freiburg, Germany). Densitometric analysis of Western blot images was performed using ImageJ 1.47v software (National Institutes of Health, Bethesda, MD, USA).

### Chromatin immunoprecipitation

Chromatin immunoprecipitation (ChIP) was performed as previously described [55]. Briefly, SW480 cells were grown in 15 cm cell culture dishes to subconfluency and treated with 25 μM LY or vehicle for 12 hours. Experiments were performed twice on different days and finally analyzed as duplicates. To crosslink transcription factors and chromatin, cells were fixed in 1% formaldehyde (Merck) in cell culture medium for 10 minutes. Chromatin bound β-Catenin was precipitated using 5 μg mouse anti-β-Catenin antibody (clone 14; BD Biosciences) and 5 μg normal mouse IgG1 (SCBT) as control. Quantitative PCR analysis of precipitated chromatin was done on a Lightcycler LC480 instrument (Roche) employing SYBR green PCR mastermix (Applied Biosystems, Carlsbad, CA, USA) and primers spanning the *Axin2* WNT responsive element (*Axin2-WRE*) and the human acetylcholine receptor promoter (*AchR*) as control region. The enrichment was expressed as percentage of the input DNA for each condition. To calculate the binding to the DNA region analyzed, the enrichment using the anti-β-Catenin antibody compared to the isotype control IP is expressed as percent of the input as described in detail by Hahn et al [55]. Primer sequences are listed in the supplementary table S4.

### Immunofluorescence microscopy

Cells were seeded at a density of approx. 80% confluence on glass cover slips in a 6 cm diameter cell culture dish. After 12 hours, medium was replaced with fresh medium containing indicated inhibitor or vehicle. After 30 min, cells were transfected with the given plasmids. When needed, the medium was replaced after 4 hours with fresh medium containing 4-OHT or ethanol respectively, together with the inhibitors or vehicle as given. After 24 hours, cover slips were removed, washed in cold PBS, fixed 15 min. with 4% paraformaldehyde / PBS, washed, permeabilized for 10 min. with 0.1 % TritonX-100 and blocked for 1 hour in FBS. The remaining cells in the culture dish were processed for Western blot analysis. Cover slips were incubated with primary antibody diluted 1:100 in 50 % FBS / PBS / 0.05 % Tween-20 for one hour, washed and incubated with Alexa546 or Alexa488 labeled secondary antibody (dilution 1:500, Invitrogen, Freiburg, Germany) for 30 min. After washing, cover slips were mounted on a glass slide using Prolong Gold antifade mounting medium (Invitrogen). Images were acquired on a Leica SP5 confocal laser scanning microscope using a 630× oil immersion objective (Leica, Wetzlar, Germany).

### Colon cancer specimens

Archival formalin fixed paraffin embedded (FFPE) tissue of 110 patients that underwent surgical resection of right sided colon cancer (CC) at the University Hospital of Munich was obtained. As a case-control study design, 55 patients without metastatic spread (M0) were matched to 55 cases in which distant metastasis to the liver had occurred (M1), with respect to tumor grade (G), T category and tumor localization. Clinicopathological data was obtained from the Munich Cancer Registry (MKR). The study was performed according to the recommendations of the local ethics committee of the Medical Faculty of the Ludwig-Maximilians Universität München.

### Immunohistochemical staining and scoring

For the analysis of PTEN expression, the monoclonal mouse anti-human PTEN specific antibody clone 6H2.1 (DAKO, Hamburg, Germany) was used at a dilution of 1:30. The specificity and reproducibility of PTEN expression testing using this antibody was previously assayed [56]. For the examination of PTEN-immunohistochemistry a score developed by Loupakis et al. was applied [57]. The intensity of cytoplasmic staining (0 - no, 1 - weak, 2 - moderate, and 3 - strong staining) were summarized to the score for the percentage of positive cells (0 - less than 25%, 1 - 25% to 50%, 2 - more than 50% positive staining cells). Specimens were defined as positive if the total score was 4 or greater. β-Catenin expression was detected using prediluted anti-β-Catenin mouse monoclonal antibody (clone 14; Ventana Medical Systems, Oro Valley, AZ, USA) as primary antibody. Whole mount tissue slides were stained on a Ventana Benchmark XT autostainer using the XT UltraView diaminobenzidine kit (Ventana Medical Systems). A staining score for nuclear expression of β-Catenin was exclusively based on the percentage of stained tumour nuclei throughout the whole tumor tissue. The scoring system was as follows: 0: negative, 1 + : <30%, 2 +: 30-60%, 3 + : >60% positive cells. Subsequently, the cases were classified into low- (scores 0 and 1) and high-grade (scores 2 and 3). To exclude non-specific staining, system controls were included in all staining runs. Evaluation of immunostaining for all markers was performed double-blinded by two pathologists (T.K.and J.N.).

### PIK3CA mutation analysis

Tumor tissue to be extracted was marked on H&E stained slides and subsequently extracted from the consecutive unstained dewaxed serial sections using scalpel blades under microscopic control. DNA was isolated from the resulting tissue using DNA-Micro-Amp® Kits (Qiagen, Hilden, Germany) following the manufacturer's instructions. *PIK3CA*-mutations were analyzed using pyro-sequencing. Briefly, Hotstar Taq-polymerase (Qiagen, Hilden, Germany) was used together with 1×PCR buffer (1.5 mM MgCl_2_), 200 µM dNTPs and 400 nM primers applying optimized PCR conditions. PCR products were analyzed using Pyro-Gold kits (Qiagen, Hilden, Germany) together with 3 nM of the corresponding sequencing primer (Table 2) on the Biotage™ Q24 device (Qiagen, Hilden, Germany). Finally, data were analyzed applying the PyroMark™ Q24 software (Qiagen, Hilden, Germany). Primer sequences and analyzed sequences as well as detailed immunohistochemistry protocols can be provided upon request.

### Statistical analyses

The significance of correlations of the parameters analyzed was calculated applying Pearson's χ^2-^test using SPSS version 20.0 (SPSS Inc., Chicago, IL, USA). For all tests a p-value lower than 0.05 was considered as statistically significant.
